# Functional partitioning through competitive learning

**DOI:** 10.3389/frai.2025.1661444

**Published:** 2025-11-05

**Authors:** Marius Tacke, Matthias Busch, Kevin Linka, Christian Cyron, Roland Aydin

**Affiliations:** ^1^Institute of Material Systems Modeling, Helmholtz-Zentrum Hereon, Geesthacht, Germany; ^2^Institute for Continuum and Material Mechanics, Hamburg University of Technology, Hamburg, Germany

**Keywords:** partitioning, clustering, unsupervised learning, machine learning, competitive learning

## Abstract

Datasets often incorporate various functional patterns related to different aspects or regimes, which are typically not equally present throughout the dataset. We propose a novel partitioning algorithm that utilizes competition between models to detect and separate these functional patterns. This competition is induced by multiple models iteratively submitting their predictions for the dataset, with the best prediction for each data point being rewarded with training on that data point. This reward mechanism amplifies each model's strengths and encourages specialization in different patterns. The specializations can then be translated into a partitioning scheme. We validate our concept with datasets with clearly distinct functional patterns, such as mechanical stress and strain data in a porous structure. Our partitioning algorithm produces valuable insights into the datasets' structure, which can serve various further applications. As a demonstration of one exemplary usage, we set up modular models consisting of multiple expert models, each learning a single partition, and compare their performance on more than twenty popular regression problems with single models learning all partitions simultaneously. Our results show significant improvements, with up to 56% loss reduction, confirming our algorithm's utility.

## 1 Introduction

Datasets can include multiple sections that adhere to distinct regimes. For instance, in stress-strain tests of materials, the initial phase exhibits elastic behavior, which is reversible. However, if the material is stretched further, it enters a phase of plastic behavior, resulting in permanent changes. Similarly, self-driving cars face unique challenges when navigating construction zones, which may be specific to certain regions of the parameter space, just as they do on highways or country roads. This mixture of functional patterns affects how difficult datasets are for models to learn. Typically, the more diverse the patterns within a dataset, the more challenging it is for a model to achieve high accuracy. In this work, we present a novel partitioning algorithm that detects such functional patterns and, when possible, separates them.

Given these mixed regimes, the modeling task can be viewed in two steps: first, split the domain, then build a model that covers all parts. In practice, these steps are often implemented within a single process, but they can also be separated. The first step—standalone domain splitting—is known as clustering. A classic example is k-means (Macqueen, 1967). Most clustering methods group points by an assumed similarity measure. In k-means, spatial proximity defines similarity. K-means iterates between assigning each point to its nearest centroid and updating centroids to the mean of assigned points. Extensions include fuzzy c-means for soft assignments (Dunn, 1974) and game-based k-means, which strengthens competition among centroids for samples (Rezaee et al., 2021). Clustering has been extensively studied; the surveys by (Jain 2010), (Du 2010), (Aggarwal and Reddy 2013), and (Ezugwu et al. 2021) provide broader overviews.

A classical approach that unifies domain splitting with modeling is the mixture of experts (MoE), introduced by (Jacobs et al. 1991). In MoE, a gating network makes soft partitions of the input space and routes samples to local experts. Training is often carried out with the expectation maximization (EM) algorithm. The latent responsibilities decouple gate and expert updates and induce competitive learning, so experts that better explain a sample are rewarded and specialization emerges. The hierarchical MoE by (Jordan and Jacobs 1994) extends this idea with tree-structured gating, which increases modularity and enables progressively refined splits. Subsequent work explored localized gates based on Gaussian densities, which yield analytic updates for the gate and faster training while preserving competition among experts (Xu et al., 1994). To manage overfitting and model complexity, variational and Bayesian formulations place distributions over parameters, improving regularization and model selection while maintaining competitive allocation of data (Waterhouse et al., 1995; Ueda and Ghahramani, 2002). Stability in multiclass settings has been analyzed, and remedies such as small learning rates and expectation conditional maximization (ECM) style separate updates have been shown to sustain specialization despite parameter coupling (Chen et al., 1999; Ng and McLachlan, 2004). Beyond neural experts, MoE has been combined with support vector machines (SVMs) and Gaussian processes (GP), including a mixture of GP experts that assign regions of the input space to different GP components. These combinations improve flexibility and scalability for nonstationary data (Meeds and Osindero, 2005; Yuan and Neubauer, 2008; Lima et al., 2007; Tresp, 2000). Extensions to time series and sequential data augment gates and experts with temporal structure and allow partitions to evolve over time (Weigend et al., 1995; Chen et al., 1996). For an accessible orientation to developments over the past two decades, see the survey of (Yuksel et al. 2012). (Shazeer et al. 2017) provided a recent efficiency proof by realizing conditional computation at scale. They introduced sparsely gated MoE layers with thousands of feedforward experts and routed only a few per example, which yielded very large capacity at modest computational cost and state-of-the-art results in language modeling and machine translation.

Beyond the classical MoE approach, several ensemble methods pursue localization and specialization without a gating network. The self-organizing map by (Kohonen 1990) uses competitive learning to arrange prototypes on a low-dimensional lattice, which promotes local specialization and is widely used for clustering and visualization. Iterative splitting methods repeatedly partition the dataset and spawn new models when accuracy remains insufficient, so experts emerge that specialize on different regions (Gordon and Crouson, 2008). (Zhang and Liu 2002) introduced the one prototype take one cluster paradigm (OPTOC), which creates models as needed and lets them compete for data points, and (Wu et al. 2004) adapted it to gene expression clustering.

There is fast-growing work on sparse MoE for large language models (LLMs) that aims to expand capacity without increasing compute per token. As one example, (Do et al. 2025) study routing in transformer-based MoE and propose USMoE that compares token choice and expert choice. Building on (Shazeer et al. 2017), (Fedus et al. 2022) integrate MoE into the transformer with a switch feedforward layer, enabling many more parameters at modest per-token compute. Refining this method, (Pham et al. 2024) address expert collapse and routing imbalance with winner-takes-all competition based on actual expert activations and with a separate router trained to predict these outcomes, which improves routing and representation diversity. For first-stage retrieval, (Guo et al. 2025) combine specialized lexical, local, and global matching experts with competitive training to balance effectiveness and efficiency.

Beyond applications, two recent theoretical studies develop mathematical foundations for MoE, analyzing when they succeed on clustered tasks and linking EM training to mirror descent with convergence guarantees (Kawata et al., 2025; Fruytier et al., 2024). (Li et al. 2022) explore feature-level rather than sample-level MoE using soft subspace clustering to assign features to multiple specialists rather than clustering samples. (Cortés et al. 2025) apply multiple choice learning with a winner takes all loss to time series forecasting, and (Nikolic et al. 2025) use sparse MoE variational autoencoders to study unsupervised specialization. (Piwko et al. 2025) propose hellsemble, an ensemble that partitions data by difficulty and trains specialists on progressively harder subsets. The sequential variant follows a fixed order and passes misclassified instances forward, while the greedy variant selects at each step the model that yields the largest validation gain. In contrast to our approach, hellsemble is largely sequential, with later models correcting earlier errors, whereas our experts operate fully in parallel. (Krishnamurthy et al. 2023) show that classical MoE can yield unintuitive and imbalanced decompositions when the gate and the experts are trained jointly, which weakens specialization. They address this with attentive gating that leverages expert outputs and with data-driven similarity regularization to encourage balanced routing, an important issue they pursue along a different path than we do. (Eigen et al. 2013) study deep MoE with two expert layers and a gating network for each layer, showing that the layers specialize in different aspects while keeping a fixed expert set and joint training. In another line of work, (Oldfield et al. 2024) design a generic MoE block that can be integrated into diverse architectures and that remains fully differentiable, using dense input-dependent routing rather than discrete selection to make the component plug and play. Finally, (Ukorigho and Owoyele 2025) present a competition-based model discovery approach close in spirit to ours, where models compete for data points and the winner discourages others from capturing similar samples to sharpen specialization. Key differences to our work include how the number of models is chosen, since they keep adding models while validation loss improves, whereas we add and drop models using explicit criteria based on the hardest samples and redundancy among specialists. Another difference is the training schedule, as they couple routing and expert optimization, while we separate partitioning because this partitioning enables a wide range of other uses, with analysis of regimes and active sampling as two examples. Furthermore, they evaluate on structurally different tasks than we do.

We propose an alternative to the classical mixture of experts: in our approach, multiple models compete for each data point. The model with the most accurate prediction is rewarded with training on that point, which drives specialization. The resulting expert preferences define a partitioning of the dataset. In this paper, we use that partitioning to build a modular model with one expert per region, and we compare it to a single global model.

Methods such as the iterative splitting of (Gordon and Crouson 2008) and the hellsemble framework of (Piwko et al. 2025) organize competition rather in a sequential split and refine loop than in parallel. While they are highly valuable for growing models, they actually react to residual error and capacity limits as the specialization they induce follows difficulty rather than stable semantic regimes. Our goal is different. We aim to expose regimes that arise from how different learners naturally win on different subsets. A central difference to classical MoE is our two-step design. We first partition the dataset, then we learn the final experts on the induced regions. This separation gives wide freedom in how to design the partitioning. In the present work, the partition is driven purely by competition. Compared to (Ukorigho and Owoyele 2025), we use this freedom to establish flexible adding and dropping modules that adjust the number of experts automatically. The framework also allows for great flexibility regarding the model class hyperparameter settings within the competition.

The partitioning we obtain enables multiple secondary uses, such as facilitating data analysis or enabling efficient sampling strategies. Consider a scenario where sampling is expensive because each data point requires a costly experiment. One could collect data in batches and rerun the partitioning after each batch. After training a separate expert for each region, regions with underperforming experts could be interpreted as harder and thus prioritized for additional sampling. This approach aligns with the paradigm of active learning, where models are deliberately exposed to data points they are most uncertain about in order to improve their weaknesses. Our approach, however, inverts this idea. In our competition-based design, models do not train on their weaknesses but on their strengths: they are rewarded with training on those data points they predict most accurately. This deliberate choice drives specialization and induces the resulting partitioning of the dataset. Rather than seeking to reduce uncertainty, we exploit certainty to create a structured division of the data, which can then support downstream tasks such as expert modeling or targeted analysis.

## 2 Materials and methods

### 2.1 Partitioning algorithm

The objective of our approach is to detect functional patterns in datasets and separate them in case they appear separable. To achieve this, we propose competition among multiple models. We intentionally refer to models in a general sense, as our approach is not limited by the type of model used. However, for simplicity, one might consider simple feedforward networks as an example. The models compete for data points, which requires them to specialize in certain functional patterns of the dataset. This specialization can be translated into a partitioning of the dataset.

Given the dataset:


$$D={\left\{\left(x_{i},y_{i}\right)\right\}}_{i=1}^{n},$$

we assume that the input features *x*_*i*_ and the output labels *y*_*i*_ are known. However, we assume that both the number of partitions and the location of their boundaries are unknown. We start with *K* models in the competition: Let $f_{\theta_{k}}:X\to \mathbb{R}$ denote the *k*-th model prediction, parameterized by θ_*k*_, where θ_*k*_ represents the set of model parameters (e.g., weights and biases):


$$f_{\theta_{k}}\left(x\right),\;k=1,\ldots ,K.$$

For each data point in the dataset, all models submit their predictions. The prediction error for each model and data point is calculated like this:


$$e_{i,k}={\left(y_{i}-f_{\theta_{k}}\left(x_{i}\right)\right)}^{2}.$$

Each data point is assigned to the model whose prediction is closest to the true value, formally expressed as:


$$a\left(i\right)=\arg\underset{k\in \left\{1,\ldots ,K\right\}}{\min}e_{i,k},$$

thereby also defining the subset of the dataset assigned to each model:


$$D_{k}=\left\{i|a\left(i\right)=k\right\}.$$

As a reward for providing the most accurate prediction, the winning model is allowed to update its parameters using this subset of data points for one training epoch. The corresponding mean squared error, which in the case of neural networks is backpropagated through the network for optimization, is defined as:


$$L_{k}\left(\theta_{k}\right)=\frac{1}{\left|D_{k}\right|}\underset{i\in D_{k}}{\sum }{\left(y_{i}-f_{\theta_{k}}\left(x_{i}\right)\right)}^{2}.$$

The global mean squared error can be expressed as:


$$L\left(\Theta \right)=\frac{1}{K}\overset{K}{\underset{k=1}{\sum }}L_{k}\left(\theta_{k}\right),$$

**Figure 1:**
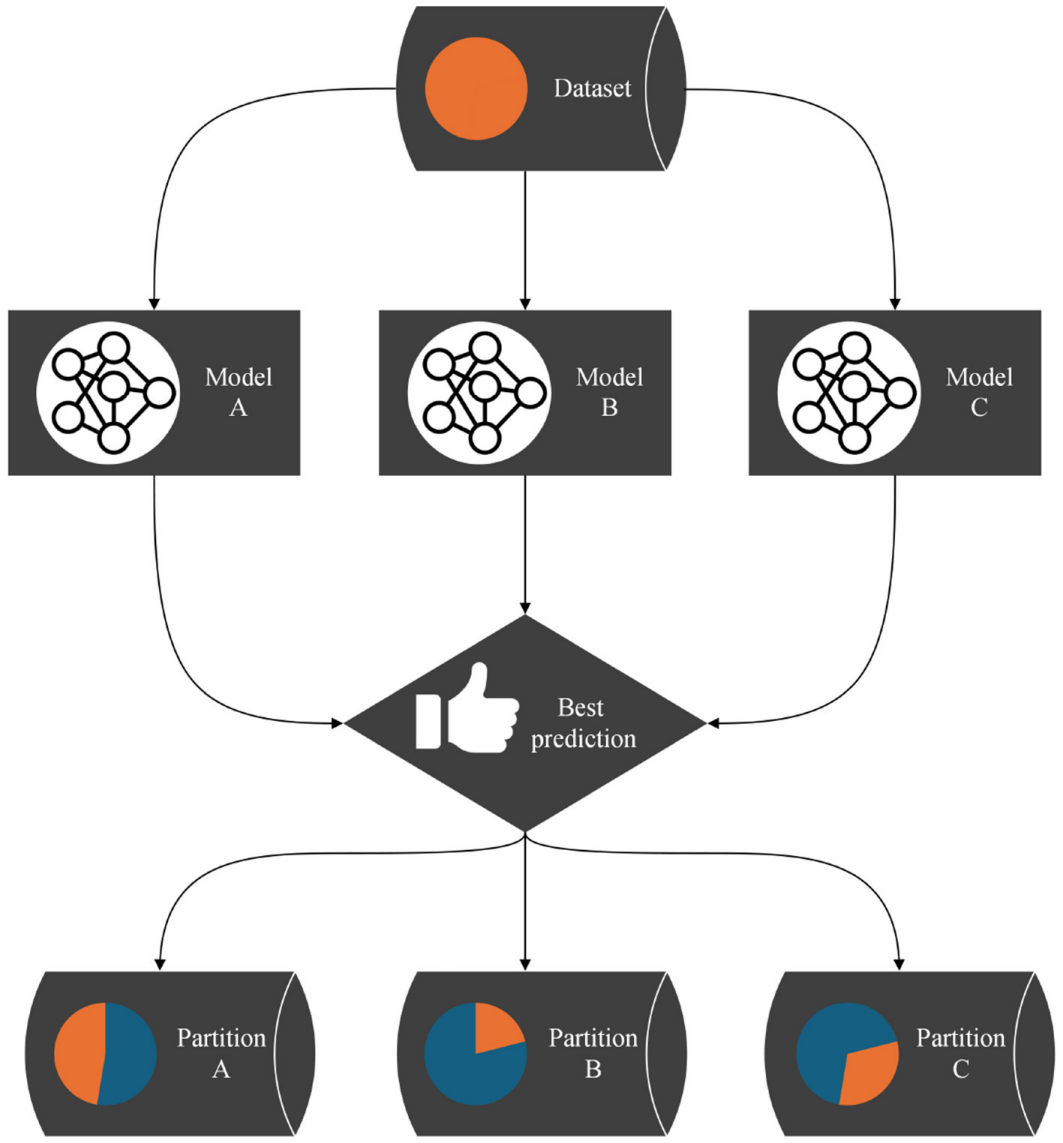
Flow chart of the partitioning algorithm: each data pointed is assigned to the model that submitted the best prediction. All models are trained with the data points in their partition for one epoch. This process is iterated.

However, this global loss is not used for optimization, as there is no trainable gating mechanism; instead, the partitioning of the dataset emerges from the competitive interaction among the networks. Algorithm 1 describes the implementation of this idea. A corresponding flowchart is shown in Figure 1.

**Algorithm 1:** Partitioning: best predictions are rewarded with training.

procedure main
for each *epoch* **do**
for each *model* **do**
Submit predictions for all data points.
end **for**
for each *datapoint* **do**
Rank models according to their predictions.
end **for**
for each *model* **do**
Train for one epoch with all won data points.
end **for**
end **for**
end **procedure**

**Figure 2:**
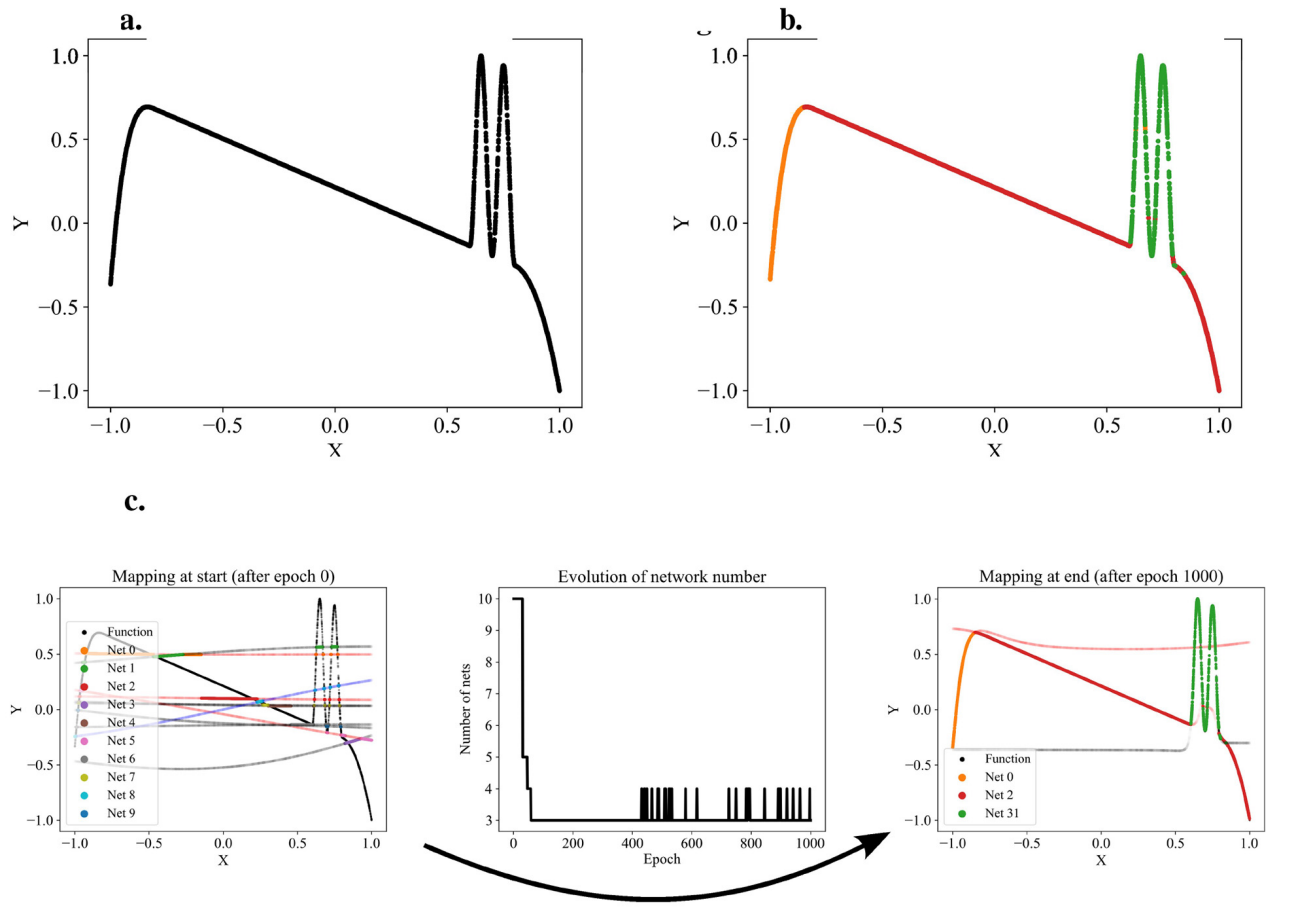
Exemplary partitioning. **(a)** Presents the self-designed test dataset, while **(b)** displays an exemplary partitioning result. **(c)** Illustrates the partitioning process, transitioning from networks with initial random predictions to the orange, red, and green networks each capturing distinct patterns. The process involves adding and removing networks as patterns are identified or networks deemed redundant.

This process—models submitting predictions, ranking the predictions, and training the models on the data points for which they provided the best predictions—is iterated. We call one such iteration an epoch of the algorithm. As the models specialize, we expect the assignments of data points to models to stabilize: a specialized expert will usually submit the best predictions for its domain. After a predefined number of epochs, the assignments of data points to models are considered final. Each model's won data points translate to a separate partition of the dataset. The hyperplanes between the partitions are stored in a support vector machine (SVM), making the partitioning technically available for other applications. Snapshots of the application of the algorithm to a one-dimensional function that we designed as a test dataset are shown in Figure 2. The transition from random predictions at the beginning to specialized experts at the end is clearly visible. The assignments of data points to the specialized experts are translated into the final partitioning.

**Figure 3:**
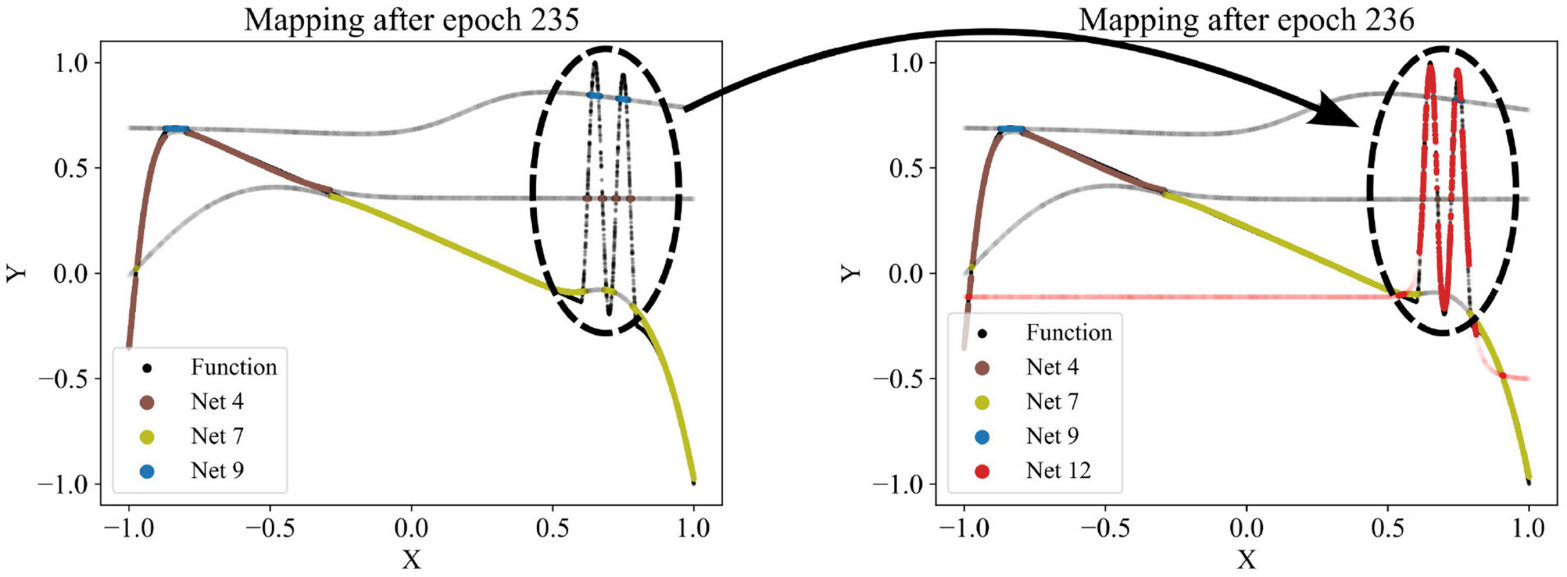
Adding a new network (red network 12) to the competition. Regularly, a new network is trained using the data points with the poorest predictions at that time. If the new network improves the overall loss, it is added to the competition. Here, the red network 12 is the first to capture the sinusoidal pattern.

**Figure 4:**
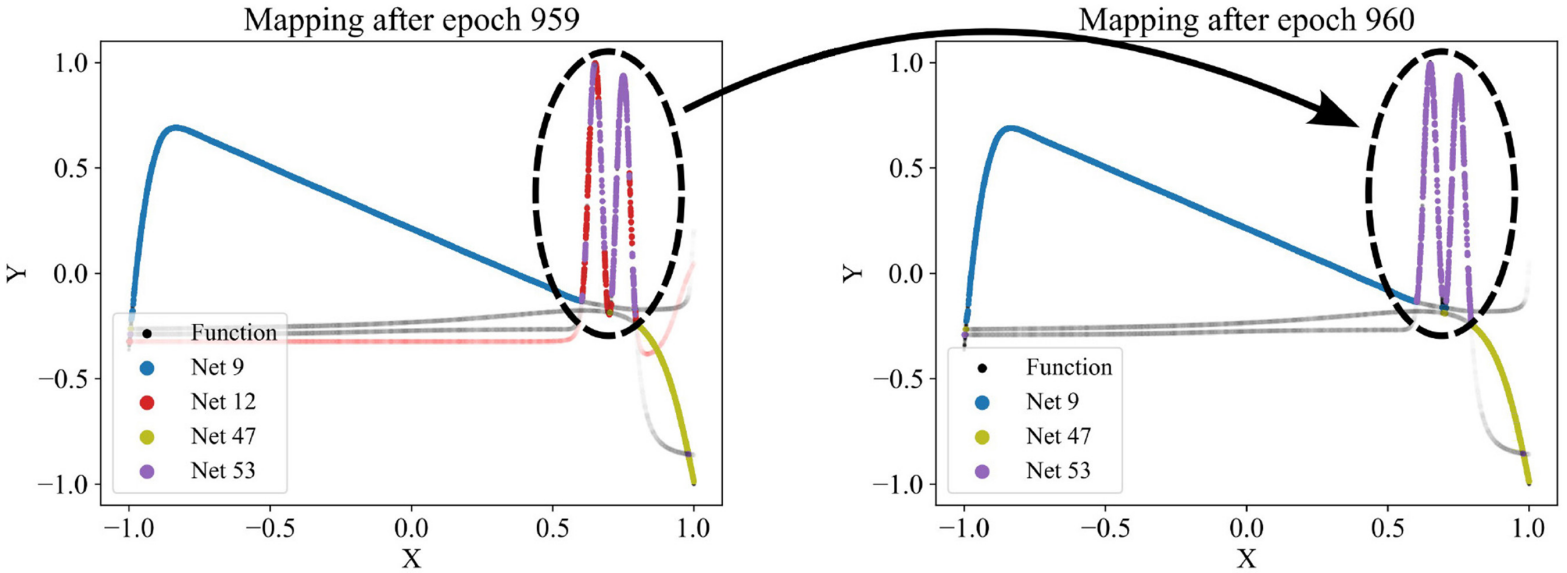
Dropping a network (red network 12) from the competition as it appears redundant, failing to capture any patterns uniquely. Regularly, for each model, we check how much the overall loss would increase if the network were removed. If the increase is small, the corresponding network is considered redundant and is discarded. Here, the red network's predictions were too similar to the purple network's predictions.

Since the number of partitions is usually unknown beforehand, the partitioning algorithm includes an adding and a dropping mechanism to dynamically adapt the number of competing models to the dataset. To evaluate whether a new model should be added to the competition, we regularly identify the data points with the poorest predictions in the dataset and train a new model on these points. The new model is added to the competition in case that improves the overall loss. Figure 3 demonstrates the addition of a model that successfully captures a significant portion of the sinusoidal section of a test function, which had previously been unlearned. For more details, see the pseudo-code of the adding mechanism in Appendix Algorithm 2. Conversely, redundant models that do not uniquely capture their own pattern should be eliminated. Such redundancy is indicated by models not winning any data points or by their predictions significantly overlapping with those of other models. The degree of redundancy is assessed by the increase in overall loss if the model were deleted. This factor is regularly checked, and all highly redundant models are removed. Figure 4 demonstrates the removal of the red model, as it only captures data points similarly well as the purple model. Appendix Algorithm 3 provides the corresponding pseudo-code. The adding and dropping mechanism are designed to balance each other. Figure 2 shows exemplary how the number of competing models is adapted to the dataset from initially ten to finally three. This process involves both adding new models to capture previously unlearned patterns and removing redundant ones.

A significant asset of our partitioning algorithm is its ability to extend to a pattern-adaptive model type, architecture, and hyperparameter search without incurring additional costs. So far, competing models have been considered similar in terms of their type, architecture, and hyperparameter settings. However, all three can be randomly varied among the models, as it is reasonable to assume that different patterns may require, for example, wider neural networks or smaller learning rates. Consequently, the algorithm's output can not only be a partitioning but also an optimal configuration of model type, architecture, and hyperparameters for each partition.

### 2.2 Modular model

**Figure 5:**
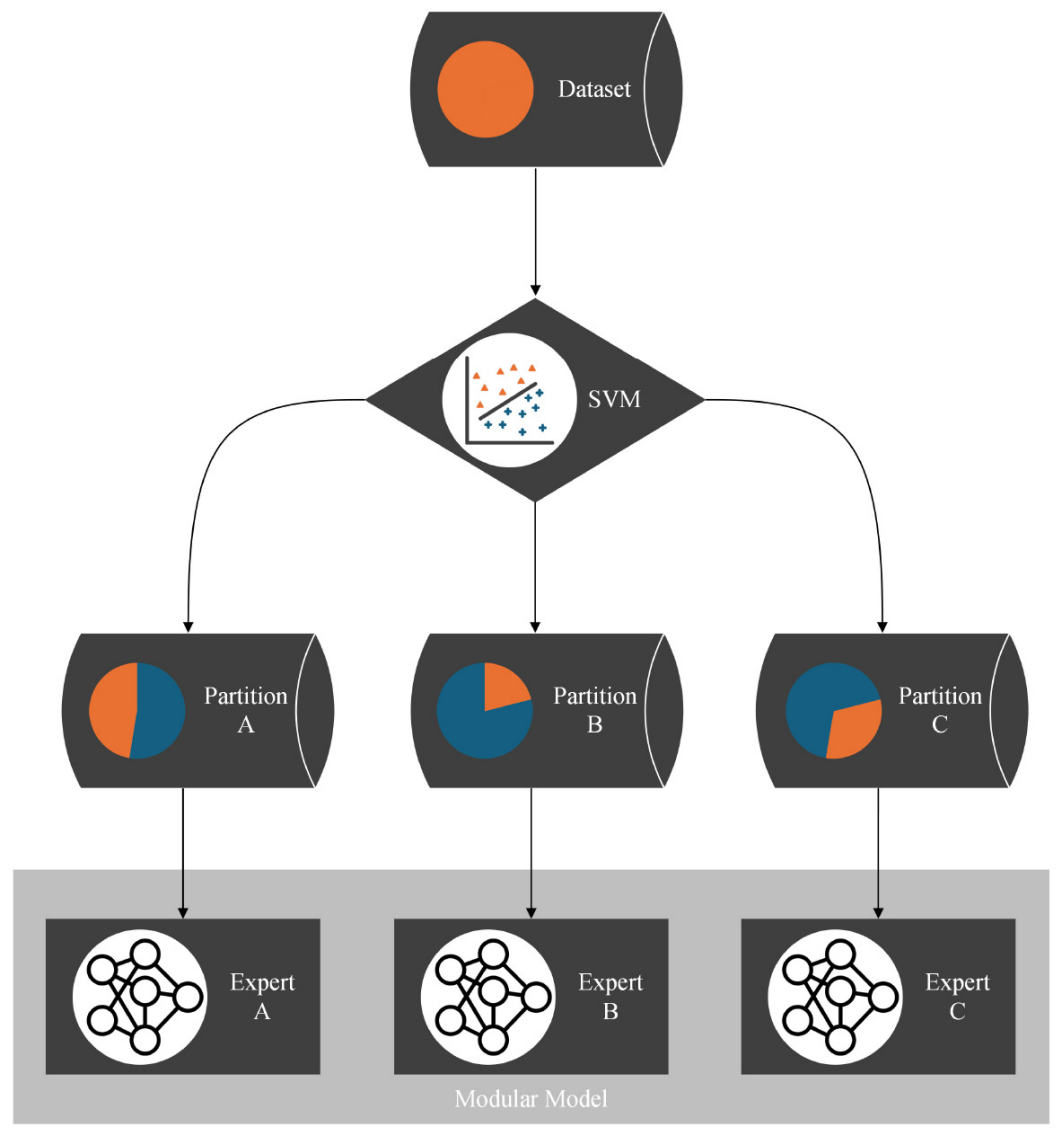
Flow chart of the modular model: each partition is learned by a separate expert model. For each data point, the SVM as a result of the partitioning algorithm decides which expert to train or to test. This way, the experts are combined to a modular model.

Applying the partitioning algorithm to datasets reveals interesting and valuable insights about the dataset's structure, as illustrated in Figure 2. Additionally, the partitioning can be utilized for various other purposes, such as learning the dataset using a divide-and-conquer approach. Traditionally, the entire dataset is used to train and optimize a single model. However, if the partitioning algorithm detects distinct functional patterns, it may be beneficial to have multiple expert models, each learning only one pattern, instead of pressing all patterns into a single model. Therefore, multiple expert models that each learn one partition are combined into a modular model. The SVM, which incorporates the boundaries between the partitions, serves as a switch between the experts. For each data point, the SVM decides which partition it belongs to and, consequently, which expert model to train or test. The structure of the modular model is illustrated with a flowchart in Figure 5. With this approach, we believe that we can reduce model complexity and increase model accuracy for datasets that are structured by multiple distinct functional patterns with little overlap.

To evaluate this approach, we compared the performance of a single model trained on the entire dataset with that of a modular model comprising multiple expert models. We speak of models in general, as the type of model can be varied. In our experiments, we used feedforward neural networks. To ensure a fair comparison, we allowed the single model to have as many trainable parameters (weights and biases) as the combined total of all experts in the modular model. We conducted a hyperparameter optimization for each expert and separately for the single model serving as the baseline. To keep the hyperparameter search space manageable, we limited the search to the most influential parameters and applied reasonable constraints: the number of layers was varied between 2 and 6, the number of neurons per layer between 4 and 10, and the learning rate between 0.0001 and 0.005. All other hyperparameters were fixed at values listed in Appendix Table 2. Within this reduced search space, we performed 100 grid search trials for each expert model and each single model. This process ensures that any advantages or disadvantages are not due to unfitting parameters or outliers. To estimate the stability of both approaches, we repeated each run, which—partitioning the dataset, training the modular model including hyperparameter optimization, and training the single model including hyperparameter optimization - ten times.

### 2.3 Datasets

**Figure 6:**
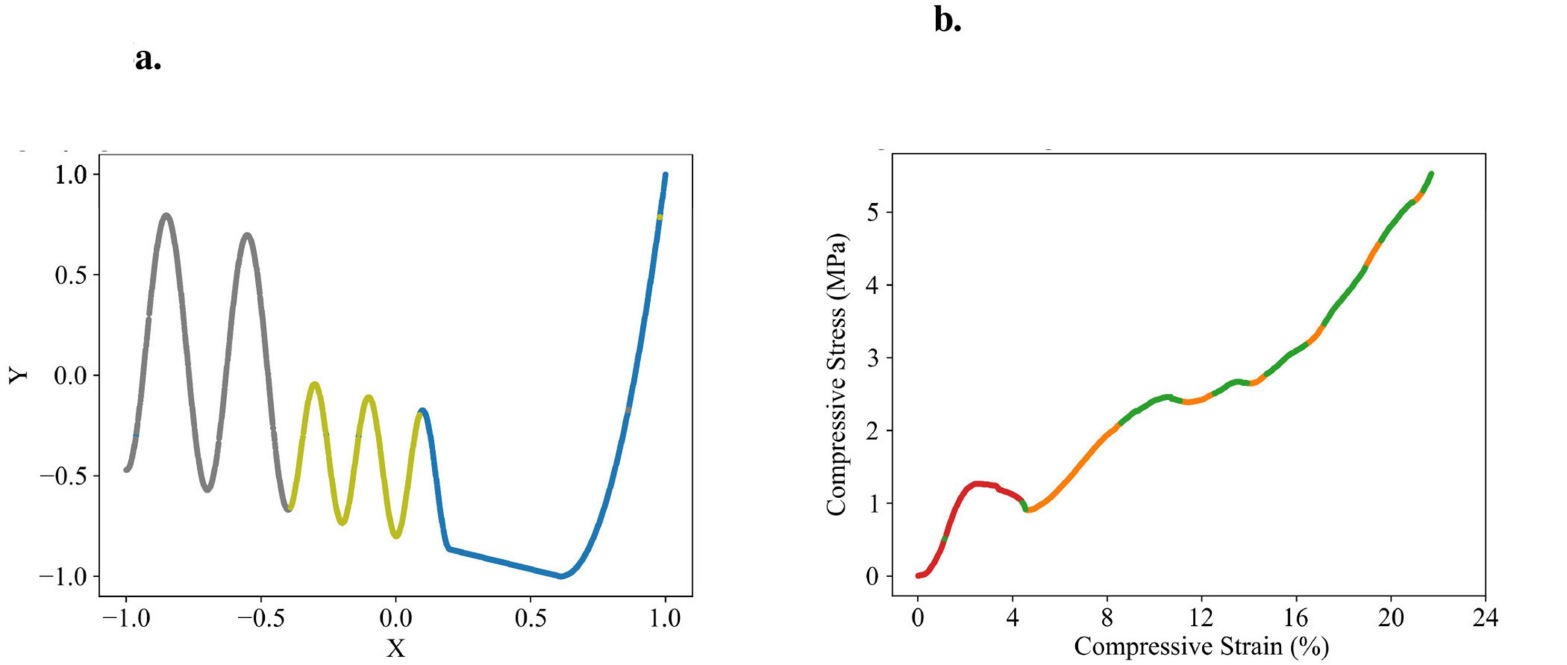
Datasets to test the partitioning algorithm, illustrated with exemplary partitioning results. **(a)** Self-designed wave-climb function with three patterns identified by the algorithm (gray, green, blue). **(b)** Porous structure's stress-strain dataset generously provided by (Ambekar et al. 2021) with three patterns identified by the algorithm (red, green, orange).

We designed one-dimensional, section-wise defined functions to serve as test datasets for validating the effectiveness of our approach and its implementation. The anomaly-crest function is illustrated in Figure 2a, and the wave-climb function is depicted in Figure 6a. Due to their section-wise definition, these functions exhibit different local functional patterns, akin to several engineering problems. One such example is modeling the stress-strain curves of materials with porous structures. These materials offer an excellent balance between weight and strength, but their stress-strain curves are typically challenging to model due to the presence of diverse functional patterns. An exemplary stress-strain curve for such a material is shown in Figure 6b. The data for this porous structure's stress-strain curve were generously provided by (Ambekar et al. 2021), who collected them. We have observed a high robustness of our partitioning approach to variations in the models random initializations. Figures 2, 6 illustrate typical results.

In addition to the one-dimensional datasets, we evaluated our method using 22 popular higher-dimensional real-world datasets from the UCI Machine Learning Repository (Kelly et al., 2024). Our tests focused exclusively on regression problems, though our approach can be readily extended to classification problems. Acknowledging that our assumption of distinct and separable characteristics may not apply to all datasets, we tested these 22 additional datasets to assess the frequency and extent to which the modular model, based on the partitioning algorithm, outperforms a single model (Imran et al., 2020; Cortez et al., 2009; Nash et al., 1995; Palechor and la Hoz Manotas, 2019; Schlimmer, 1987; Cortez and Morais, 2008; Feldmesser, 1987; Yeh, 2018; E and Cho, 2020; Tsanas and Xifara, 2012; Yeh, 2007; Tfekci and Kaya, 2014; Cortez, 2014; Quinlan, 1993; Matzka, 2020; Wolberg et al., 1995; Fernandes et al., 2015; Janosi et al., 1988; Tsanas and Little, 2009; Chen, 2017; Moro et al., 2016; Hamidieh, 2018). A characterization of all test cases is provided in Appendix Table 3.

## 3 Results

We evaluated the predictions of both approaches using mean squared error (MSE) and R^2^. We expected our pipeline of partitioning algorithm and modular model to outperform the single model in some, but not all test cases. This was confirmed: the pipeline showed clear advantages in 6 out of 25 cases. For the two synthetic test functions, the modular model outperformed the single model by orders of magnitude, validating the concept. On the porous structure's stress-strain data, which inspired the test functions, the modular model reduced the test MSE by 54% on average over 10 runs. The modular model also showed strong performance on three real-world datasets. On the energy efficiency dataset, it achieved a 56% reduction in test MSE, on the automobile dataset, 29%, and on the student performance dataset, 10%, all averaged over 10 runs.

**Figure 7:**
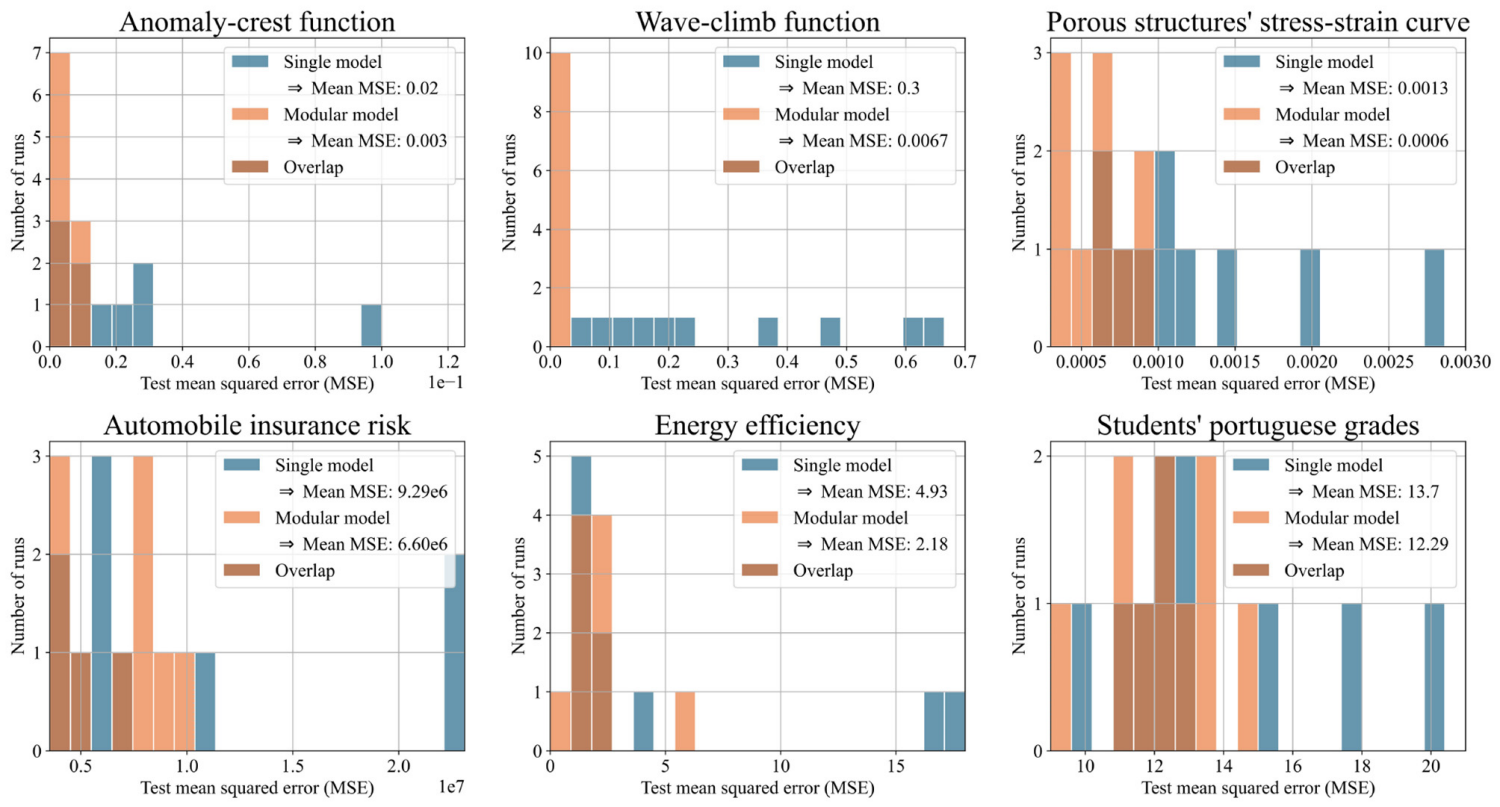
Histograms illustrating the test mean squared error (MSE) of single and modular model for ten runs with each of the six selected datasets. The higher the bars on the left side, the better the performance.

**Figure 8:**
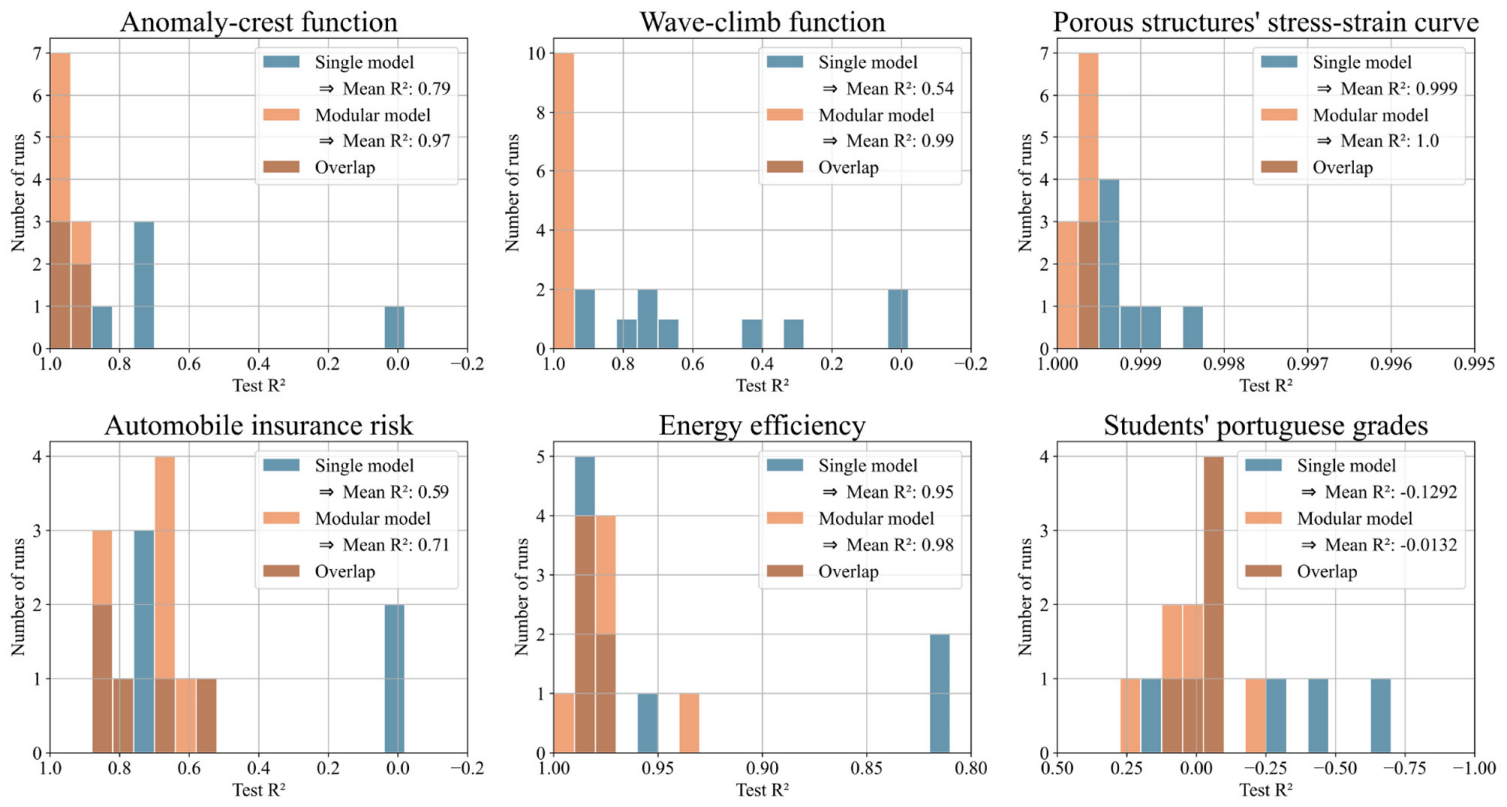
Histograms illustrating the test R^2^ scores of single and modular model for ten runs with each of the six selected datasets. The higher the bars on the left side, the better the performance.

Figure 7 shows histograms of test MSE for the modular and single models. Figure 8 shows the same predictions evaluated with R^2^, offering a more intuitive illustration of the performance. Both figures focus on the six datasets where the modular model had a significant advantage. Each histogram displays results from ten runs per model. The x-axis shows either test MSE or R^2^; the y-axis shows the number of runs achieving each value. Higher bars on the left indicate better performance.

Table 1 summarizes the six datasets shown in the histograms, listing features, labels, and data points. Appendix Table 3 includes this information for all 25 datasets and is placed in the appendix due to its length.

**Table 1:** Characterization of the six datasets in Figures 7 and 8.

**Dataset**	**URL**	**Synthetic**	**# features**	**# labels**	**# samples**
Anomaly-Crest function	URL	Yes	1	1	10,000
Wave-climb function	URL	Yes	1	1	10,000
Automobile insurance	URL	No	25	1	205
Energy efficiency	URL	No	8	2	768
Students' grades	URL	No	30	1	649
Stress-strain curve	URL	No	1	1	4,065

**Figure 9:**
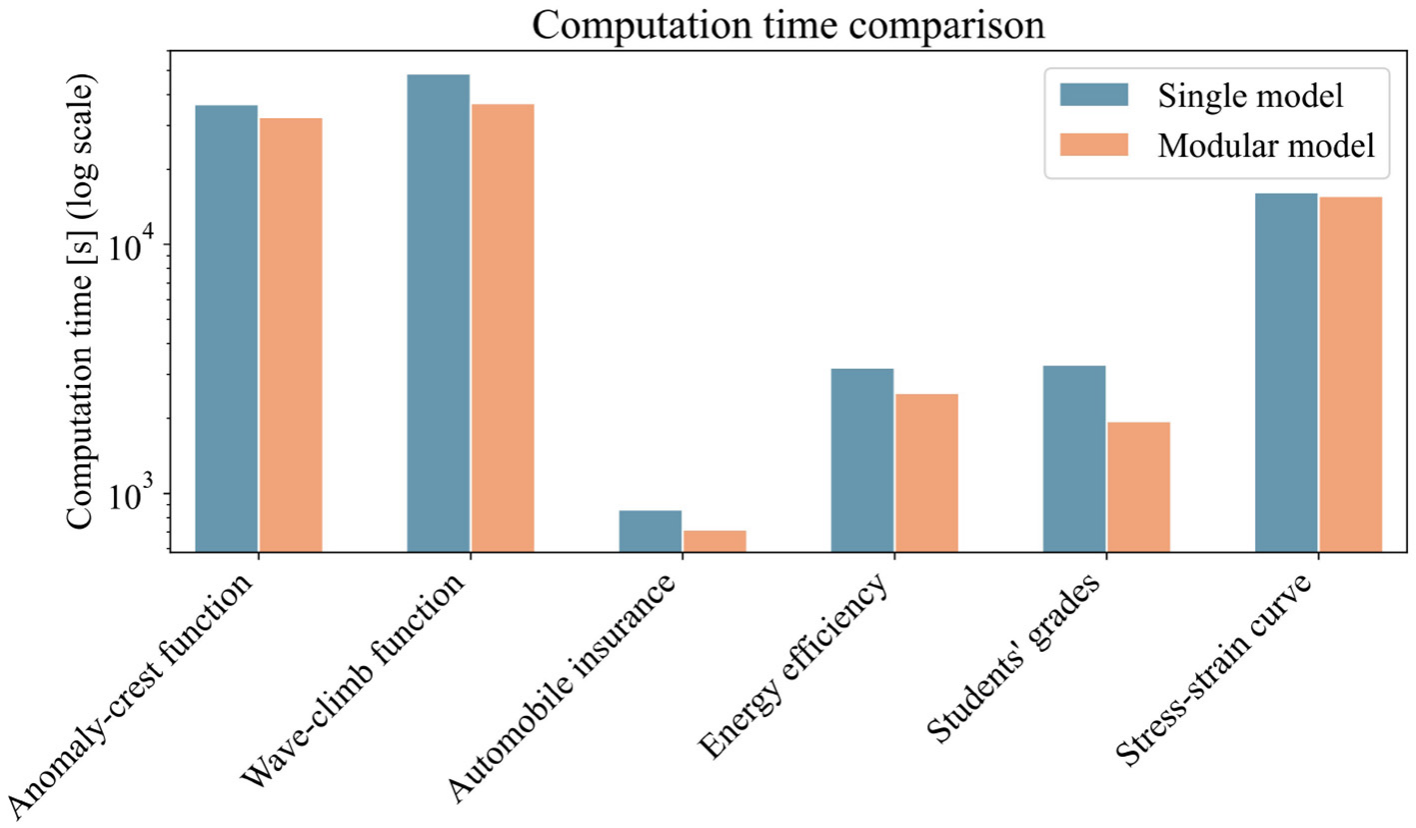
Bar plot showing the computation time for the training of both the single-model and modular-model approaches. For the modular model, the total time includes the execution of the partitioning algorithm. For both approaches, the time required to perform a 100-trial grid search for hyperparameter optimization is included.

Since training efficiency is key in machine learning, we also compared the training times of the modular model and single model approach. We measured the time required for a 100-trial grid search for hyperparameter tuning. For the modular model, we also included the time to run the partitioning algorithm. Figure 9 presents a bar plot of training times for the six highlighted datasets. The x-axis shows the datasets, the y-axis (log scale) shows computation time in seconds on a standard desktop computer (Intel Core i9-11950H @ 2.60GHz, 64GB RAM, NVIDIA RTX A5000 with 24GB VRAM). Compared to the hyperparameter search, the partitioning algorithm adds negligible time. More impactful is the modular model's use of multiple but smaller models, which speeds up backpropagation. Overall, training times are similar, with a slight advantage for the modular model.

## 4 Discussion

As introduced in Section 2.1, the partitioning algorithm is based on the competition between multiple models: iteratively, each model is trained on the data points for which it provided the best predictions. The progressive reinforcement of each model's strengths drives their specialization, which we exploit to partition the dataset. Through competition, the models naturally align with distinct functional patterns in the data.

The application of our partitioning algorithm to the anomaly-crest function demonstrates that the competition between multiple models is generally effective for developing specialized experts and separating different functional patterns. The primary value of this partitioning lies in its ability to detect these distinct patterns and provide insights into the dataset's structure. For the anomaly-crest function, the four identified sections clearly differ in their functional characteristics (see Figure 2). In the case of the wave-climb function, the algorithm successfully separates the two sinusoidal sections with different frequencies and amplitudes, as well as a final u-shaped section, which seems reasonable (see Figure 6a). For the porous structure's stress-strain dataset, it is noteworthy that the first hook is identified as a distinct pattern. Subsequently, all sections with concave curvature are captured by the green model, while all sections with convex curvature are captured by the orange model. This partitioning was surprising, but it appears that the models find it easier to learn either concave or convex curvatures exclusively (see Figure 6b). The models themselves detecting which functional patterns can be learned well coherently was exactly what we were aiming for.

One potential concern is that a single model might, due to a lucky initialization, dominate the competition and suppress the emergence of specialized models. On the one hand, our adding mechanism tackles this by relying on relative performance: new networks are iteratively trained on the samples with the least accurate predictions. Because this threshold is relative to the best models performance rather than absolute, a newly initialized model, trained specifically on challenging samples, can always outperform the current model and enter the competition. That said, we do not claim that every dataset can be effectively partitioned using our approach. Some datasets exhibit a single coherent pattern or contain overlapping patterns that resist separation. If a single model consistently outperforms others, it may simply reflect that the dataset is best modeled holistically. In such cases, we view it as a strength of our method that it naturally converges to a single domain, signaling to the user that partitioning is not beneficial and that a unified model may be more appropriate. This behavior aligns with our results: while the modular model was not superior across all datasets, it outperformed the single model on six out of the 25 datasets tested. For the porous structure's stress-strain dataset and the energy efficiency dataset, the modular model achieved a loss reduction of over 50% (see Figure 7). These findings support our hypothesis that for datasets with separable patterns, specialized expert models can offer significant advantages over a single unified model.

Even in cases where the modular model achieves lower average loss than the single model, the histograms show that individual trials can still favor the single model. This variability arises in part from randomness in the partitioning process: although the algorithm tends to converge to similar partitions, some runs produce splits that are more effective than others. More importantly, both approaches are influenced by stochastic factors during training, such as initialization and sample shuffling, which naturally lead to performance variance. Additionally, since we are dealing with standard feedforward neural networks and standard optimization algorithms, we also encounter standard challenges, such as models getting stuck in local minima, which can affect individual outcomes.

In Appendix Section 1, we describe a detailed analysis of the factors contributing to the performance of the modular model. Our findings reveal a correlation between the number of patterns identified by the partitioning algorithm and the modular model's performance: the more distinct patterns in the dataset, the better the modular model performs relative to the single model. This aligns with our expectation that not all datasets are suitable for our approach. The partitioning algorithm should primarily be applied to datasets that are expected to exhibit predominant patterns with minimal overlap. The clearer the patterns, the more effective the modular model is expected to be. Additionally, we examined the impact of our pattern-adaptive hyperparameter search, which optimizes the hyperparameter settings for each pattern. We discovered that tailoring the learning rates to each partition enhances the modular model's performance. However, our results indicate that adjusting the numbers of layers and neurons per layer for each pattern does not provide any significant advantage. Finally, we aimed to verify that the partitioning algorithm identifies substantial patterns rather than merely separating small and challenging snippets. Our results confirm that the more homogeneous the partition proportions, the more successful the modular model tends to be.

While this study exclusively uses feedforward neural networks, our framework is not limited to this model type. Since competition is moderated solely by prediction accuracy, the approach is flexible enough to incorporate a wide range of models, from simple linear regressors to very complex architectures such as large language models (LLMs). This generality opens up opportunities for future experiments with diverse model types, depending on dataset characteristics.

There are numerous potential applications of our partitioning, many of which we may not have yet considered. We found it important to illustrate a path that leads to measurable improvements by leveraging our partitioning results. One application we plan to explore in the future is using the partitioning algorithm for active learning. In the context of expensive data points, the following data collection loop could be advantageous: first, collect a batch of data points; then, apply the partitioning algorithm; and finally, train each partition with a separate model, akin to the modular model approach. Instead of immediately combining their predictions, we could assess each expert's performance and adjust the collection of new data points accordingly. Partitions that are more challenging to learn should receive more data points, while easier partitions should receive fewer. This approach could lead to a more efficient use of the data point budget. The process can be repeated iteratively. For instance, with a budget of 500 data points, we could run this process 10 times, each time distributing 50 data points according to the difficulty of the experts in learning their partitions in the last iteration.

## 5 Conclusions

In this paper, we introduced a novel partitioning algorithm. To the best of our knowledge, this algorithm is unique in its use of competition between models to generate a general-purpose partitioning scheme, without constraints on the dataset's origin or order. The partitioning is achieved by having multiple models iteratively submit their predictions for all points in the dataset and being rewarded for the best predictions with training on the corresponding data points. This process induces specialization in the models, which is then translated into a partitioning.

We demonstrated that our algorithm is both widely applicable and useful. Its wide applicability was shown by valuable results across datasets of varying dimensionalities, sparsities, and contexts—from student education to engineering stress-strain tests. The utility of our algorithm was illustrated in two primary ways: first, the partitioning inherently provides insights into the dataset's structure. For instance, three distinct patterns were detected in the porous structure's stress-strain dataset: an initial hook, convex, and concave parts. Second, certain datasets can be learned more accurately with a modular model based on our partitioning algorithm than with a single model. If a model's accuracy in learning a dataset is unsatisfactory and the dataset is likely structured along predominant patterns with little overlap, we recommend applying our pipeline of the partitioning algorithm and modular model. Particularly in the context of expensive data points, improving the model on this path without adding more data points can be financially beneficial. In the future, we will explore a third application: adapting data collection strategies based on our partitioning algorithm.

## Data Availability

The data for the section-wise defined functions is available at https://github.com/FunctionalPartitioning/FunctionalPartitioning. The stress-strain curve data for the porous structure is available upon request from (Ambekar et al. 2021), the original data collectors. All the high-dimensional, real-world datasets used as benchmarks to evaluate the effectiveness of our approach can be obtained from the UCI Machine Learning Repository (Kelly et al., 2024).
