# Supplementary material for: Functional partitioning through competitive learning
Source: Front Artif Intell. 2025 Nov 5;8:1661444. doi: 10.3389/frai.2025.1661444 (PMC12626917; doi:10.3389/frai.2025.1661444)
Supplement: Supplementary file 1 [file Data_Sheet_1.pdf]

1 APPENDIX

1.1 Analysis of modular model performance

We observed that, for several datasets, the modular model utilizing the partitioning algorithm significantly outperformed the single model. To analyze these observations in more detail, we created the plots shown in Figure 8. We compared the performance of the modular and single models across ten test runs for each of the 25 datasets. The datasets with final losses displayed in the histograms in Figures 7 and 8 are marked with unique colors for identification, while all other datasets are illustrated in orange.

Figure 8. Evaluation of the influence of multiple characteristics of the modular model on the performance of the modular model compared to the single model across all tested datasets.

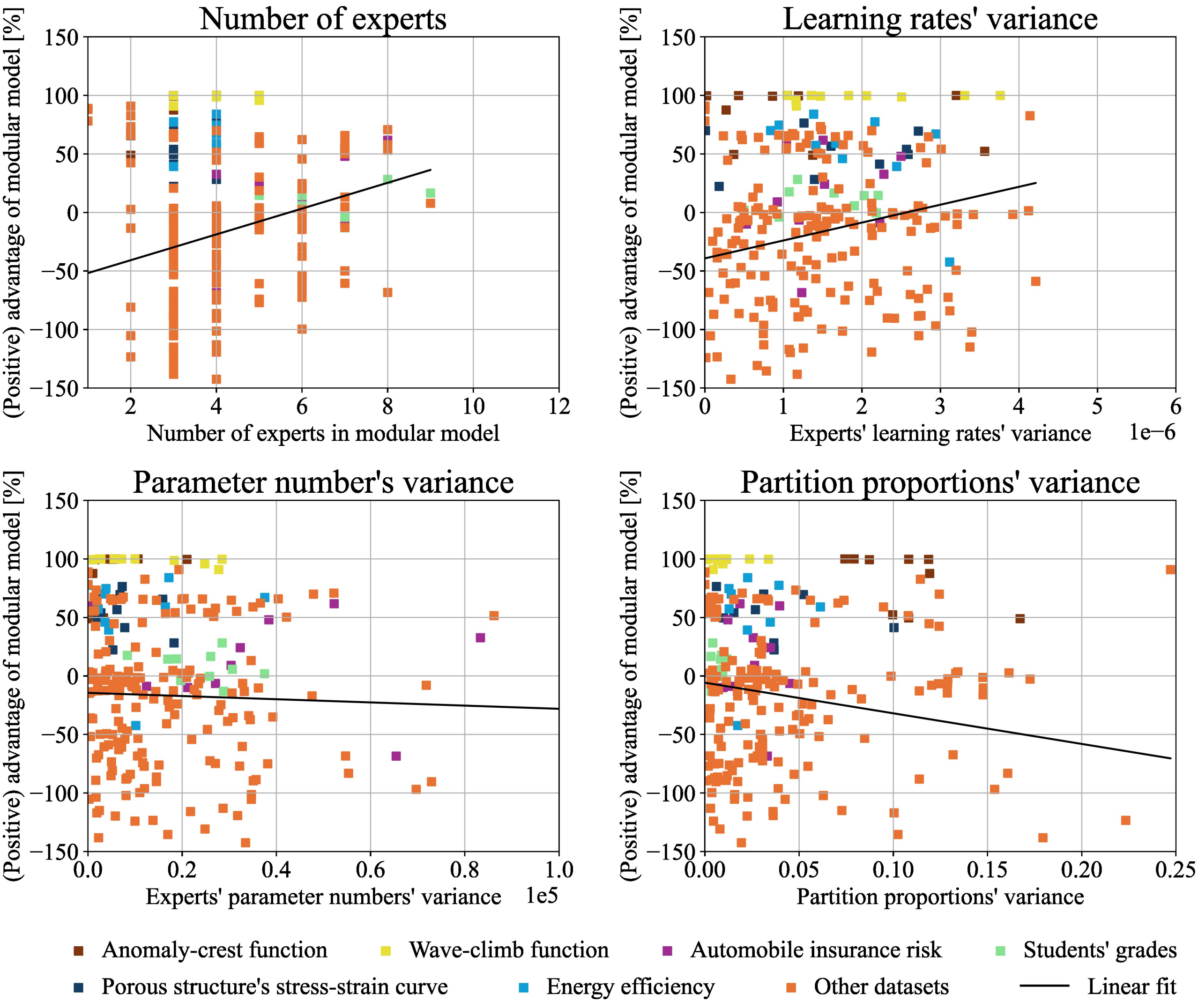

For each dataset, we computed the mean test loss of the single model over the ten test runs. We then compared the test losses of the modular model against this benchmark. For example, if the single models achieved an average loss of 100 and the modular model achieved a loss of 80, we recorded a performance value of 20%. Conversely, if the modular model achieved a loss of 120, we recorded a performance value of -20%. These performance measures were plotted against potentially influential parameters: the number

of experts, the variance in the experts' learning rates, the variance in the experts' parameter counts, and the variance in the partition proportions.

Firstly, we observed that the performance of the modular model compared to the single model improves with an increasing number of experts (see Fig. 8). Since the number of experts in the modular model corresponds to the number of patterns the partitioning algorithm has separated, this insight is more about the datasets that work well with this approach than about the modular model itself. The more separate patterns are found within one dataset, the better the modular model can be expected to work. Given our initial expectation that not all datasets would contain separable patterns, this finding is not surprising. The more clearly a dataset is structured around separable patterns, the more effective our approach appears to be.

The modular model allows for the adjustment of hyperparameters locally for each expert, unlike the single model with a global uniform hyperparameter setting. In our experiments, we varied only the learning rate, the number of layers, and the number of neurons per layer. For this analysis, we combined the number of layers and the number of neurons per layer into a single metric: the number of trainable parameters. We evaluated the impact of locally adapting the hyperparameter settings to each pattern. The more the hyperparameter settings are tailored to each pattern, and the more they differ from a constant setting for the entire dataset, the greater their variance among all experts in a single run. Consequently, we plotted the performance of the modular model compared to the single model versus the variance in experts' learning rates and the variance in experts' trainable parameters (see Fig. 8). We observed a moderate correlation between the modular model's performance and the adaptation of learning rates, but no correlation with the adaptation of trainable parameters. Notably, also with small variances in learning rates, modular models outperformed single models. We conclude that locally adapting learning rates to each pattern is moderately beneficial, whereas adjusting the number of layers and neurons per layer does not appear to have a significant impact.

Finally, we plotted the performance of the modular model compared to the single model against the variance in partition proportions for each run (see Fig. 8). Our aim was to verify that the algorithm identifies significant patterns rather than just isolating small, difficult segments. Our findings confirm this hypothesis, indicating that the more uniform the partition proportions, the more effective the modular model becomes.

1.2 Adding and dropping mechanisms in detail

For those interested in understanding the partitioning algorithm in full detail, this section provides the pseudo-code for the adding (see Alg. 1) and dropping (see Alg. 2) mechanism of the partitioning algorithm. Additionally, Table 2 lists all significant hyperparameter settings for both the partitioning algorithm and the modular model.

Algorithm 1 Adding: train new model with badly predicted data points.

```
procedure ADDMODEL
    allLosses ←Losses of best prediction for each data point
    lossBound = mean(allLosses) + std(allLosses)
    dataPoints ←Data points with loss above lossBound
    oldLoss ←Mean loss of dataPoints
    newModel = new Model()
    newModel.train(dataPoints)
    newLoss = newModel.getLoss()
    if newLoss < oldLoss then
        add(newModel)
    end if
end procedure
```

Algorithm 2 Dropping: drop highly redundant models.

```
procedure DROPMODELS
    for each dataPoint do lossWithAllModels += lossOfBestModel
    end for
    for each model do
        for each dataPoint do
            if model == bestModel then
                lossWithoutModel += lossOfNextBestModel
            else
                lossWithoutModel += lossOfBestModel
            end if
        end for
        replacability = lossWithoutModel/lossWithAllModels
        // 10% greater loss without model → replacability = 1.1
        if replacability < droppingReplacability then
            drop(model)
        end if
    end for
end procedure
```

1.3 Details on hyperparameter search and test cases

Table 2. Hyperparameter settings of the partitioning algorithm and the single and modular model during the experiments.

Optimizer	Adam
Activation function	tanh
Epochs partitioning algorithm	1,000
Epochs modular model	500
Scaled feature range	[-1,1]
Batch size	16
Partitioning: initial model number	10
Partitioning: adding check	every epoch
Partitioning: dropping check	every epoch
Partitioning: dropping threshold	1.8
Hyperparameter search runs	100
Minimal layer number	2
Maximal layer number	6
Minimal neuron number per layer	4
Maximal neuron number per layer	10
Minimal learning rate	0.0001
Maximal learning rate	0.005

Table 3. Characterization of all tested datasets.

Dataset	URL	Synthetic	# features	# labels	# samples	Significant improvement
Anomaly-Crest function	URL	Yes	1	1	10,000	Yes
Wave-climb function	URL	Yes	1	1	10,000	Yes
Garment employees	URL	No	14	1	1,197	No
Red wine	URL	No	11	1	4,898	No
Abalone	URL	No	8	1	4,177	No
Obesity	URL	No	16	1	2,111	No
Automobile insurance	URL	No	25	1	205	Yes
Forest fires	URL	No	12	1	517	No
Computer hardware	URL	No	9	1	209	No
Real estate valuation	URL	No	6	1	414	No
Seoul bike sharing	URL	No	13	1	8,760	No
Energy efficiency	URL	No	8	2	768	Yes
Concrete strength	URL	No	8	1	1,030	No
Power plant	URL	No	4	1	9,568	No
Students' grades	URL	No	30	1	649	Yes
Automobile MPG	URL	No	7	1	398	No
Maintenance	URL	No	6	6	10,000	No
Breast cancer	URL	No	33	1	198	No
News popularity	URL	No	58	1	39,797	No
Heart disease	URL	No	13	1	303	No
Parkinson	URL	No	19	2	5,875	No
Bejing	URL	No	11	1	43,824	No
Facebook	URL	No	18	1	500	No
Superconductivity	URL	No	81	1	21,263	No
Stress-strain curve	URL	No	1	1	4,065	Yes
